# Schleichender demografischer Wandel und neurologische Rehabilitation – Teil 2: Handlungsmöglichkeiten

**DOI:** 10.1007/s00115-022-01416-w

**Published:** 2023-01-11

**Authors:** Stefan Knecht, Harmut Reiners, Mario Siebler, Thomas Platz, Agnes Flöel, Reinhard Busse

**Affiliations:** 1 Institut für Klinische Neurowissenschaften, AG Neurorehabilitation, Heinrich-Heine Universität Düsseldorf, Düsseldorf, Deutschland; 2 Referat Grundsatzfragen der Gesundheitspolitik, Ministerium für Arbeit, Soziales, Gesundheit und Familie (MAGS) Brandenburg, Potsdam, Deutschland; 3 Fachklinik für Neurologie, Rhein/Ruhr, Essen, Deutschland; 4 Institut für Neurorehabilitation und Evidenzbasierung, An-Institut der Universität Greifswald, BDH-Klinik Greifswald, Greifswald, Deutschland; 5 AG Neurorehabilitation, Universitätsmedizin Greifswald, Greifswald, Deutschland; 6 Klinik und Poliklinik für Neurologie, Universitätsmedizin Greifswald, Greifswald, Deutschland; 7 Management im Gesundheitswesen, Fakultät Wirtschaft und Management, Technische Universität Berlin, Berlin, Deutschland

**Keywords:** Anschlussrehabilitation, Fachkräftemangel, Digitalisierung, Beatmungsentwöhnung, Pflege, Post acute care, Skilled labor shortage, Medical information technology, Weaning, Skilled nursing

## Abstract

In seinem jetzigen Zustand wird das deutsche Gesundheitssystem nicht in der Lage sein, eine ansteigende Zahl alter Menschen in der Bevölkerung mit einer verminderten Zahl qualifizierter Erwerbspersonen zu versorgen. Diese Problematik betrifft besonders die postakute Versorgung von Schwerkranken, wie in einem ersten von zwei Beitragsteilen unter Situationsbeschreibung dargelegt wurde. Hier diskutieren wir Perspektiven und Handlungsmöglichkeiten. Eine führende Schlussfolgerung ist, dass akut- und rehabilitativmedizinische Maßnahmen deutlich wirksamer als bisher auf einander abgestimmt werden könnten und sollten.

## Abstract (en)

In its current state the German healthcare system will not be able to adequately care for a growing proportion of older patients with a decreasing healthcare work force. This is particularly so in the postacute care of severely ill patients. In a second of two parts we discuss the perspectives and options at hand. A major conclusion is that substantial gains could be obtained by regulatory adjustments that better align acute care and rehabilitative measures.

## Einleitung

In 20 Jahren müssen ein Drittel mehr alte Menschen mit einem Siebtel weniger Erwerbspersonen versorgt werden. Dies ist mit den derzeitigen Strukturen unseres Gesundheitssystems nicht möglich. Das Schleichende der Entwicklung ist Segen und Fluch zugleich: Einerseits könnten wir uns weitblickend vorbereiten. Andererseits verleitet der langsame Verlauf aber zu Bequemlichkeit und einem Es-wird-schon-werden-Denken. Die Eigendynamik unseres Gesundheitssystems im bisherigen Regulationsrahmen jedoch war dysfunktional. Von Krankheit betroffene Menschen verlieren Chancen auf Rückgewinnung von Selbstständigkeit. Zusätzlich sind Ausgaben und Personalbindung unnötig gestiegen. Diese Zusammenhänge haben wir in einem vorangehenden Beitragsteil unter *Schleichender demografischer Wandel und neurologische Rehabilitation – Teil 1: Situationsbeschreibung* dargelegt. In diesem zweiten Beitragsteil, bemühen wir uns, Handlungsoptionen wie auch deren Grenzen aufzuzeigen.

## Personal

Eine in den nächsten 20 Jahren nötige Personalaufstockung von über 200.000 Mitarbeiter*innen allein in der Pflege in Zeiten einer rückläufigen Erwerbsbevölkerung ist nicht allein durch Geld zu bewerkstelligen. Der demografische Wandel in Deutschland und anderen Industrienationen betrifft alle Wirtschaftszweige. Arbeitgeber beklagen bereits jetzt ein Fehlen von Facharbeiter*innen, Ingenieur*innen, Informatiker*innen und anderen Akademiker*innen [3]. Als einzelne Gesundheitseinrichtung kurzfristig gegen andere Wirtschaftszweige oder untereinander über Löhne oder andere Annehmlichkeiten zu konkurrieren entspricht einer Auktion, bei der nicht die Zahl der Güter, sondern nur der Preis steigen kann.

Das aus den DRGs ausgegliederte Pflegebudget bei stationären Leistungen erleichtert jedoch politische Mechanismen für Lohnerhöhungen im Pflegeberuf insgesamt und so zumindest mittelfristig für eine Anreizsteigerung gegenüber anderen Branchen. So könnten Kräfte aus sich strukturell verändernden Wirtschaftssektoren für das Gesundheitssystem gewonnen werden. Derzeit erfahren z. B. die über 3 Mio. Beschäftigten des Verkaufsbereichs massive Veränderungen ihres Berufsfeldes durch den Onlinehandel. Gleichzeitig gibt es medizinisch ausgebildete oder in Ausbildung befindliche Menschen im Ausland, die zur Immigration bereit wären. Genügend Bewerber*innen für das Medizinstudium gäbe es ohnehin.

Diese Gruppen müssen allerdings über einen langen Zeitraum fachlich oder sprachlich qualifiziert werden. Angesichts der schleichenden Entwicklung wäre dies jedoch möglich, vorausgesetzt man wird früh und umfassend aktiv. Die größte Wirksamkeit könnte über eine staatliche oder andersartige gemeinschaftliche Initiative erreicht werden. Denn Einzelakteure handeln und planen meist zu kurzsichtig und individual-rational. Unterstützt werden könnte eine Qualifikationsinitiative durch „massive open online courses“, getragen z. B. durch das Bundesgesundheitsministerium.

Leider ist in dieser Richtung bisher keine oder nur wenig Aktivität zu verzeichnen. Angesichts des notwendigen Vorlaufes von mehreren Jahren und des Abfalls der Erwerbspersonenkurve ist daher eine weitere Verknappung von Personal im Gesundheits- und Pflegebereich am wahrscheinlichsten. Die Auswirkungen sind schwer abzuschätzen. Möglicherweise werden Menschen, die bisher als nicht geeignet angesehen wurden, angestellt, um Minimalfunktionen aufrechtzuerhalten. Dies würde die Versorgungsqualität belasten.

## Kosten

Der Anteil der Gesundheitskosten am Bruttoinlandsprodukt wird bei uns wie auch in anderen entwickelten Ländern weiter steigen [16]. Das ist volkswirtschaftlich und politisch nicht trivial, insbesondere, wenn noch andere kostenträchtige Transformationen wie Treibhausgasreduktion nötig sind – oder vermehrte Rüstungsausgaben. Kostenverschiebungen sind normale Vorgänge. So entfiel vor 100 Jahren über die Hälfte der Lebenshaltungskosten auf Ernährung; heute sind es weit weniger als ein Zehntel. Allerdings beruhen die meisten Kostenverschiebungen auf Marktmechanismen, während über das Gewicht von Gemeingütern wie Gesundheits- und Pflegeversorgung gesellschaftlich entschieden werden muss. Entsprechende Diskussionen sind für von Wahlen abhängige Entscheidungsträger nicht die beliebtesten.

Der wichtigste Faktor zur Finanzierung steigender Gesundheitskosten in einem Sozialstaat bleibt die Erhöhung des Bruttoinlandprodukts, also der gesamtwirtschaftlichen Produktivität. Daneben können die anteiligen Zusatzbeiträge, Basisbeiträge oder der über Steuern finanzierte staatliche Zuschuss von derzeit bereits knapp 30 Mrd. € erhöht werden [22]. Es könnte auch die Finanzierungsgrundlage durch die Eingliederung der privaten in die gesetzliche Krankenversicherung erweitert werden.

Bei Kostendiskussionen kann der Frage nach Kostenbegrenzung durch Leistungsbegrenzung nicht ausgewichen werden. Augenfällige Leistungsbegrenzungen aus unmittelbar ökonomischen Gründen gab es bisher kaum. Dies ändert sich jetzt aber mit dem Auftrag des Bundesverfassungsgerichtes an den Gesetzgeber, Triagekriterien für die Intensivmedizin festzulegen (1 BvR 1541/20).

Im Einvernehmen mit Patient*innen werden Leistungen auch zum Lebensende hin beschränkt. Orientierung geben Initiativen wie „Gemeinsam Klug Entscheiden“, bei denen es um die bewusste Hinterfragung von Maximalmedizin geht [1]. Was als Lebensende wahrgenommen wird, ist jedoch subjektiv, kulturabhängig und wandelbar [6]. Schwerkranke selbst neigen dazu, sich als Last für andere wahrzunehmen und daher auf Leistungen zu verzichten [11]. Dies könnte ein Grund sein, warum das Vorenthalten von Rehabilitation bisher so klaglos verlief. Tragisch ist, dass der Verzicht auf Rehabilitation ein Sparen an der falschen Stelle ist und nur bewirkt, dass sich durch größere Pflegebedürftigkeit die Last für Betroffene, das Sozialsystem und Angehörige erhöht.

## Automatisierung

Angesichts des sich verschiebenden Verhältnisses zwischen Patient*innen und Personal fragt sich, wie weit menschliche Arbeitskraft durch Automatisierung im Gesundheitssystem eingespart werden könnte. Das Paradebeispiel der Produktivitätssteigerung durch Substitution menschlicher Arbeit ist die Autoindustrie. Dort wird pro Arbeitsstunde heute mehr als 40-mal mehr Wert geschaffen als vor 60 Jahren [17]. Vergleichende Zahlen aus dem Gesundheitssystem sind uns nicht bekannt. Wir würden aber schätzen, dass hier die Produktivitätssteigerung maximal im niedrig einstelligen Bereich liegt. (Gemeint ist hier die quantitative und nicht eine qualitative Steigerung.) Symbol für Veränderungen im produzierenden Gewerbe ist der Industrieroboter, der menschliche Arbeit – z. B. das standardisierte Schweißen in der Autofertigung – ersetzt hat. Im Gesundheitssystem liegt der größte Arbeitsblock in der Pflege. Pflegerische Kernarbeit substituierende Roboter sind derzeit nicht absehbar und dürften es aus konzeptionellen Gründen noch länger bleiben. Der zweite Zeitblock ist das Gespräch. Auch hier ist eine mehr als anteilige Digitalisierung derzeit weder erkennbar noch wünschenswert. Entsprechend wurden sogar in vielen Krankenhausbereichen Pflegepersonaluntergrenzen verpflichtend gemacht, um betriebswirtschaftlich motivierter Personalausdünnung entgegenzusteuern. Wie lange diese Maßnahme bei weiter auseinandergehender demografischer Schere aufrechterhalten werden kann, bleibt abzuwarten. Während der SARS-CoV-2-Pandemie zumindest wurde sie, gerade eingeführt, erst einmal ausgesetzt.

In der Chirurgie gibt es bereits Operationsroboter. Diese sparen aber kein Personal ein, sondern bieten nur gewisse technische Vorteile. In der Rehabilitation gibt es Therapieroboter z. B. in Form motorisierter und sensorisch geregelter Exoskelette, die das Körpergewicht von Patient*innen halten und sie beim Gehen unterstützen können. Da Patient*innen für diese Geräte aber vorbereitet und dann überwacht werden müssen, ist die Personaleinsparung eingeschränkt. Zusätzlich sind die Indikationen für diese Art des Trainings begrenzt.

In den genannten Bereichen sind technisch anspruchsvolle Interaktionen zwischen Maschine und menschlichem Körper nötig. Diese Schwierigkeit entfällt jedoch bei kognitiven Funktionen. So gibt es mittlerweile verschiedene evidenzbasierte digitale Therapien für Depressionen oder Planungsstörungen [12, 24]. Auch läuft derzeit ein multidisziplinäres Verbundprojekt zur digitalen Aphasietherapie (www.rehalingo.com). Denkbar ist ferner der Einsatz sozial interaktiver humanoider Roboter und zwar immer dann, wenn Patient*innen zwar der fachlich spezifischen und sozial interaktiven Trainingsanleitung bedürfen, jedoch nicht der physischen Unterstützung durch Therapeut*innen. Solche Systeme bieten die Chance, hohe Intensitäten spezifischer Trainingsformen ohne Erhöhung des Personalbedarfs zu realisieren. Ob dieser Ansatz in der Neurorehabilitation anwendbar ist, wird aktuell in einem Verbundforschungsvorhaben in M‑V geprüft (www.ebrain-science.de). Wenige Ansätze gibt es bisher zur digitalen Patienten- oder Angehörigenschulung [18]. Noch weniger gibt es zur digitalen Ausbildung in medizinischen Berufen, die über der SARS-CoV-2-Pandemie geschuldete Onlineveranstaltungen hinausgehen.

## Pfadeffizienz

Der Industrieroboter ist zwar das Symbol, aber eben nur das Symbol der Automatisierung. Tatsächlich trug er bloß einen begrenzten Teil zur Produktivitätssteigerung in der industriellen Fertigung bei. Ein erheblicher weiterer Teil ging auf verbesserte Pfadeffizienz zurück durch ständige datengetriebene Überprüfung von Produktionsketten und anschließendem zielgerichtetem Umbau. Dieses Vorgehen ist auch für Dienstleistungen relevant und kann deren Produktivität erhöhen [17]. Ähnlich wie bei der Automatisierung spielt hier Digitaltechnologie, also der Einsatz von Mikroelektronik, eine zentrale Rolle, weil sie erlaubt, Prozesse über Raum und Zeit hinweg sichtbar, vernetzt und steuerbar zu machen.

Das Potenzial für die Gesundheitsversorgung in Deutschland wird erahnbar, wenn man sich die bisherigen Patientenpfadinsuffizienzen anschaut: Wechseln die Behandelnden von Patient*innen, z. B. bei Abteilungs- oder Sektorenwechsel, werden Verlegungsbriefe, Befundberichte, Medikamentenlisten u. Ä. erstellt und weitergereicht – oft verzögert und unvollständig. Wenn es gut funktioniert, werden relevante Bilder mitgegeben oder auf Anforderung nachgeliefert. All diese Schritte entfallen in vollständig digitalisierten Gesundheitssystemen, bei denen alle Behandler auf dieselbe Dokumentation zugreifen und Information intelligent und strukturiert und alle Berufsgruppen einbeziehend erfasst wurde. Solch eine Struktur könnte im ambulanten Bereich gut durch verknüpfte telemedizinische Betreuungsangebote ergänzt werden.

Werden derzeit – auch ohne Behandlerwechsel – Untersuchungen angefordert oder gehen Befunde ein, erfolgt dies über zeitverzögerte Zwischenschritte statt simultan. Vitalparameter wie Blutdruck oder Temperatur werden im Normalstationsbetrieb oft noch analog erhoben und übermittelt statt über mobiles Monitoring vollständig telematisch und gegebenenfalls im klinischen Kontext, z. B. einer drohenden Sepsis, vorbewertet. Effizientere Informationserhebung, Übermittlung und Dokumentation, aber auch Aufgabendelegation könnten insbesondere in der Pflege mehr Kapazitäten für Kernaufgaben freisetzen. Bisher müssen Informationen noch zusammengesucht und abgerufen werden, statt auf einem durch Nutzer konfigurierbaren und sich selbst aktualisierendem Datenbildschirm zu erscheinen. Krankenhausinformationssysteme sind bis auf erste Medikationsprogramme noch weitestgehend passiv, statt Information auf Lücken, Plausibilitäten und Diskrepanzen zu überprüfen und Hinweise, Vorschläge oder Warnungen zu geben.

Noch effizienzhemmender als die bisherige informationelle Unübersichtlich- und Schwerfälligkeit innerhalb klinischer Abteilungen ist jedoch die Intransparenz bei der Abstimmung zwischen unterschiedlichen Akteuren des Gesundheitssystems. Es handelt sich um Probleme, die im klinischen Alltag u. a. unter den Begriff „Abtelefonieren“ fallen. Sie entstehen häufig bei Verlegungen. Ein Beispiel war die Schwierigkeit zu Beginn der SARS-CoV-2-Pandemie, für Patient*innen ein Intensivbett zu finden, weil man schlicht nicht wusste, wer ein freies Bett hatte. Durch das Intensivregister gab es hier Besserung. Es kann allerdings angenommen werden, dass entsprechende Meldelisten vielerorts manuell statt automatisiert gepflegt werden. Ähnliches gilt für poststationäre Rehabilitation oder Pflegeversorgung. Hier etablieren sich mittlerweile elektronische Matching-Plattformen. Diese sind jedoch nur begrenzt an klinische Informationssysteme angeschlossen oder ärztlich geprägt.

Durch das Fehlen einer vernetzten und durchgängigen Informationsinfrastruktur verzichten wir auch als Gesellschaft auf Überblick. Wir wissen durch das Fallpauschalensystem zwar über Mengen an Diagnosen und Prozeduren, aber kaum etwas über Pfade, Verzögerungen, Kapazitätsmängel oder mittelfristige Effekte von Therapien auf Selbstständigkeit, Pflegebedürftigkeit oder Tod.

Im Gegensatz zu vielen staatlichen Gesundheitssystemen liegt Deutschland mit seinem Bismarckschen Modell und seinen uneinheitlichen Akteuren in der institutionsübergreifenden Digitalisierung weit zurück [26]. Hier genutzte Hard- und Software ist zwar nicht perfekt, wäre aber für viele lokale Prozesssteuerungszwecke bereits tauglich. Das insbesondere für größere Vernetzung missliche Problem der Cyberkriminalität braucht jedoch aufwendige Abwehrmaßnahmen mit spezialisierten Abteilungen, Kopfgeld-Programmen u. Ä. Das wiederum setzt große Unternehmen voraus und spricht für eine staatlich verantwortete, einheitliche informationstechnologische Grundstruktur. Mit der mehrstufigen Einführung der elektronischen Patientenakte ist Deutschland jetzt aufgebrochen. Bis zu den aus klinischer Dokumentation gewonnen klinischen Registern und registerbasierten Versorgungsstudien wie in Skandinavien ist der Weg aber noch sehr weit [15]. Dies ist bedauerlich, da der demografische Wandel jetzt passiert und datenbasierte Effizienzverbesserungen so früh wie möglich realisiert werden sollten.

## Struktureffizienz

Effiziente Versorgungspfade benötigen eine passende Struktur. Hier sind wir mit dem historisch gewachsenen sektorierten deutschen Gesundheitssystem konfrontiert. Skandinavische Gesundheitssysteme, insbesondere das dänische, werden gerne als Beispiel für effiziente Medizinstrukturen angeführt und als Vorbild für Deutschland angeführt.

Die dänische Gesundheitsversorgung beeindruckt auf vielen Ebenen. Zehn Jahre hat man sich auf verschieden Ebenen über Inhalt und Art eines Umbaus verständigt und dann das System über weitere 10 Jahre strukturell umgebaut. Die mittlere Krankenhausverweildauer liegt nunmehr bei 3,1 Tagen, also weniger als halb so lang wie in Deutschland [7]. Die 30-Tage-Sterblichkeit nach akutem Herzinfarkt wurde zwischen 2006 und 2015 auf 4 je 100 Patient*innen und damit auf die Hälfte des deutschen Niveaus gesenkt [2]. Hinsichtlich der Digitalisierung gehört das Gesundheitssystem Dänemarks zu den fortschrittlichsten weltweit. Der Datenaustausch zwischen den Gesundheitsversorgern findet flächendeckend elektronisch statt. Insbesondere wird für die stationäre und niedergelassene Versorgung dieselbe elektronische Akte genutzt [26]. Dänemark verfügt wie andere skandinavische Länder und teilweise gemeinsam mit ihnen über hervorragende Datenregister als Basis für Versorgungsoptimierung (https://www.esundhed.dk/). Besonderen Widerhall fand, dass Dänemark die Zahl seiner Krankenhäuser seit 2007 halbiert hat. Die verbliebenen Krankenhäuser sind mit großen multidisziplinären Aufnahmeabteilungen ausgerüstet und teilweise mit auch außerhalb der Regelarbeitszeiten tätigen ambulanten Ärzt*innen. Eine Übernahme in Fachabteilungen erfolgt nur bei Patient*innen mit erwarteter Behandlungsdauer von über 48 h [7]. Schwerkrankenrehabilitation ist spezialisiert. Für Schädel-Hirn-Trauma-Patient*innen gibt es im Land insgesamt zwei Zentren [19]. Daneben wird Rehabilitation als Teil einer kommunalen basalen und meist nicht ärztlich betreuten Gesundheitsversorgung in Gesundheitszentren angeboten [2].

Bei den dänischen Zahlen ist zu berücksichtigen, dass das dortige Gesundheitssystem ebenso wie in anderen skandinavischen Ländern weitestgehend staatlich, einheitlich und steuergetragen ist [21]. Die Krankenhäuser versorgen 20-mal so viel ambulante wie stationäre Patient*innen. (In Deutschland haben sogar Universitätskliniken mit ihren Institutsambulanzen nur um 5‑mal so viel ambulante wie stationäre Patient*innen.) Trotz Halbierung der Zahl der Krankenhäuser zwischen 2007 und 2015 hat der Umbau des dänischen Gesundheitssystems die Zahl der Betten in diesem Zeitraum nur um 17 % reduziert. Die Zahl der Ärzt*innen hingegen ist um 19 % und die der Pflegenden um 13 % gestiegen. Die Wartezeiten für geplante Operationen lagen mit 47 Tagen im Jahr 2015 weiter sehr hoch als Hinweis darauf, dass das Verhältnis von vorgehaltenen zu benötigten Kapazitäten nicht dem entspricht, was wir in Deutschland gewöhnt sind. Daneben setzt Dänemark nach Norwegen international den zweithöchsten Anteil seiner Erwerbsbevölkerung, nämlich 17 %, im kombinierten Gesundheits- und Sozialbereich ein, Deutschland 13 % [20].

Während sich hinsichtlich der aktuellen und erwarteten zukünftigen Kosten für den Gesundheits- und Pflegebereich keine wesentlichen Unterschiede zwischen Dänemark und Deutschland finden [16], zeigen die dortigen Reformen doch, dass ein Gesundheitssystem umbaubar ist und dass mit einheitlicher Verantwortlichkeit und flächendeckender Digitalisierung ambulante, stationäre und rehabilitative Versorgung eng vernetzt werden können. Und es zeigt, dass deutlich kürzere mittlere Liegezeiten in Krankenhäusern als in Deutschland möglich sind.

Kürzere Krankenhausverweildauern erscheinen wirtschaftlich sinnvoll, weil Krankenhausbehandlung gemeinhin die teuerste Art der Gesundheitsversorgung ist. Voraussetzung ist allerdings, dass nicht an anderer Stelle Mehrkosten generiert werden – etwa durch Direktverlegungen in intensivmedizinische Pflege oder Pflegeheime. Wesentlich für die kurzen Krankenhausverweildauern in Dänemark ist die Einbettung der stationären Versorgung in Vor- und Nachbehandlung. In Deutschland gibt es auch Bemühungen in diese Richtung, u. a. durch Ambulantisierung von Krankenhausleistungen etwa über Hybridfallpauschalen, wie im Koalitionsvertrag der derzeitigen Bundesregierung skizziert. Der Ansatz besteht darin, die Versorgungskette eines Falles so zu organisieren, dass die Ressource Krankenhausbett nur genutzt wird, wenn und solange sie nötig ist. Weil die Möglichkeiten hierzu jedoch vom Einzelfall abhängen, muss das Vorgehen ärztlich organisiert und gesteuert werden.

Den Krankenhäusern in Deutschland ist durch die Logik der Fallpauschalen letztlich die gleiche Aufgabe gestellt, nämlich eine Verkürzung der Belegung von Krankenhausbetten. Nur haben sie bisher kaum Möglichkeiten, die übrige Versorgung zu organisieren und zu steuern. Dies betrifft nicht nur die ambulante Behandlung ihrer Patient*innen. Es betrifft auch die postakute Versorgung von noch nicht wieder selbsthilfefähigen Patient*innen. Mit der neurologisch-neurochirurgischen Frührehabilitation hat Deutschland zwar wie Dänemark eine Schwerpunktrehabilitation von Schwerstkranken, hat aber regional großen Bettenmangel. Zusätzlich gibt es das Problem der Anschlussrehabilitation von Patient*innen, die zwar nicht mehr engmaschig medizinisch überwacht werden müssen, aber auch noch nicht wieder selbst aufstehen und sich selbst versorgen können (Neuroreha-Phase C).

Der Gemeinsame Bundesausschuss (G-BA) hat jetzt, wie vom Intensivpflege- und Rehabilitationsstärkungsgesetz gefordert, Kriterien für eine prüfungsfreie Verlegung in Anschlussrehabilitationen vorgelegt, aber just die für nicht selbsthilfefähige Patient*innen relevante Neurorehabilitation der Phase C ausgespart, sprich für diese Gruppe die Bewilligungshürde belassen [9]. Die Gründe für dieses Politikum sind unklar. Diskutiert wird, dass die Krankenkassen über das Bewilligungsverfahren Patienten weiter in „billige“ Rehabilitationskliniken steuern wollen. Sie könnten auch beherrscht sein von der Sorge, dass Patient*innen aus Gründen der Erlösoptimierung zu früh aus dem Krankenhaus in die Rehabilitation verlegt würden [27]. Angesichts heutiger und stärker noch zukünftiger alter und multimorbider Patient*innen würden jedoch bei dieser Sicht der Dinge Patient*innen immer zu früh verlegt. Denn auch noch in der neurologischen Anschlussrehabilitation erleiden 2 von 5 Patient*innen eine oder mehrere Komplikationen [13]. Mehr als die Hälfte dieser Komplikation tritt über 60 Tage nach dem ursprünglichen Akutereignis auf. So lange kann niemand Patient*innen im Akutkrankenhaus halten, insbesondere weil sie über diese Zeit keine intensive Rehabilitation bekommen würden. Wenn Bettlägerige jedoch nicht mobilisiert werden, verlieren sie Kraft, Muskeln, Knochensubstanz sowie Koordinationsfähigkeit und erleiden häufiger Komplikationen wie Blutzuckerregulationsstörungen, Thrombosen oder Lungenentzündungen [25].

Rehabilitationskliniken müssen in der Tat stetig mehr Aufwand für die medizinische Versorgung der älter und kränker werdenden Patient*innen betreiben, der im Phasenmodell nicht abgebildet ist. Daher ist es an der Zeit, in der Rehabilitation aufwandsbasierte Vergütungen zu etablieren, die auch Morbiditätsstufen berücksichtigen – ähnlich dem PCCL (Patient Clinical Complexity Level) im Fallpauschalensystem der Krankenhäuser. Geeignete Kalkulationsgrundlagen liegen vor [5].

Dass das Bewilligungsverfahren Betroffenen, Kliniken und Kostenträgern schadet, ist mittlerweile empirisch belegt dank eines natürlichen Experimentes während der ersten Phase der SARS-Cov-2-Pandemie, als das Bewilligungsverfahren für 6 Wochen ausgesetzt wurde. Die stationäre Verweildauer im Krankenhaus verkürzte sich um 10 Tage, die stationäre Verweildauer in der Rehaklinik stieg nicht an, Patient*innen hatten nicht mehr Komplikationen, sondern erholten sich besser [23]. Ohne Bewilligungsverfahren ließe sich daher in neurologischen Kliniken die über alle stationären Patient*innen gemittelte Verweildauer von 7,6 auf unter 6,6 Tage verkürzen – auch zum Vorteil der Patienten.

## Was können Leistungserbringer tun?

Der demografische Wandel passiert nicht erst morgen, sondern ist bereits im Gang. Mittleres Alter und Komorbiditäten von Krankenhauspatient*innen sind gestiegen und steigen schleichend weiter. Dies verändert die klinische Arbeit, macht sie anspruchsvoller und aufwendiger [14]. Gleichzeitig ist Personal bereits jetzt knapp. Daher müssen auch Kliniker sich bereits heute den demografischen Veränderungen stellen.

Personal bei schrumpfender Erwerbsbevölkerung, insbesondere mehr Personal für mehr Patient*innen wird nicht von alleine an die Tür klopfen, sondern muss mit Jahren Vorlauf eingeladen werden. Diese Einladungen müssen von den Krankenhausträgern und der Politik eingefordert und eingesehen werden.

Ressourcen für Gesundheits- und Pflegeversorgung sind Zankapfel von Interessenpolitik. Eine Erhöhung des Anteils der gesellschaftlichen Aufwendung für Gesundheit ist keine hinreichende, aber eine notwendige Antwort auf die Herausforderungen durch den demografischen Wandel. Bei allen berechtigten Verteilungs- und Einspardiskussionen bleibt bestehen: Arbeit von Menschen wird im Gegensatz zu Arbeit von Maschinen nicht billiger. Da Akutmedizin, medizinische Rehabilitation und Pflege im Wesentlichen Arbeit von Menschen sind, werden wir für mehr und kränkere Patient*innen schlicht mehr Arbeit von Menschen brauchen, die mehr kosten wird. Im Gegenzug heißt das aber auch, dass Patient*innen, die keine stationären Leistungen benötigen, sondern ausschließlich ambulant behandelt werden könnten (etwa solche mit sog. „ambulant-sensitiven Konditionen“ oder mit ambulantisierbaren Operationen), auch nicht mehr stationär versorgt werden sollten, schon um das „frei“ werdende (Pflege‑)Personal für andere Patient*innen nutzen zu können. Ärzt*innen können und sollten diesen Punkt in der öffentlichen Diskussion unterstreichen. Damit können sie helfen, den notwendigen politischen Prozess zu beschleunigen.

Automatisierung und Digitalisierung zum (Teil‑)Ersatz ärztlicher, pflegerischer oder therapeutischer Kernleistungen erscheint derzeit nur begrenzt möglich, auch wenn solche Ansätze und deren Erforschung einen wesentlichen Teil der „Zukunftsstrategie“ darstellen sollten. Informationstechnologie erlaubt jedoch, Transparenz, Vernetzung und Steuerung von Abläufen zu verbessern und damit schnellere und effizientere Versorgungspfade, die so nicht nur kostengünstiger, sondern auch qualitativ besser werden können. Informatiker*innen alleine jedoch können diese Aufgabe nicht für die klinisch Tätigen leisten. Sie muss von Mediziner*innen vorangetrieben werden, die die Systeme verstehen, gestalten und nutzen.

Strukturen des Gesundheitssystems in Deutschland wird man nicht von heute auf morgen ändern – aber vielleicht auf übermorgen. Krankenhäuser können politische Ansätze zu einer strategisch wirksamen Integration von Kranken- und Pflegekassen unterstützen, damit unnötige stationäre Pflegeversorgung vermieden wird. Sie könnten ferner dahin wirken, dass Verlegungszeiten bis zu Anschlussrehabilitationen der Kassen genauso als Qualitätsparameter veröffentlicht werden wie Verweildauern oder Operationszahlen der Krankenhäuser. Dies könnte Krankenkassen zu einer Anpassung von Rehabilitationskapazitäten und Vereinbarung von Morbidität berücksichtigenden Entgelten bewegen.

**Figure 1:**
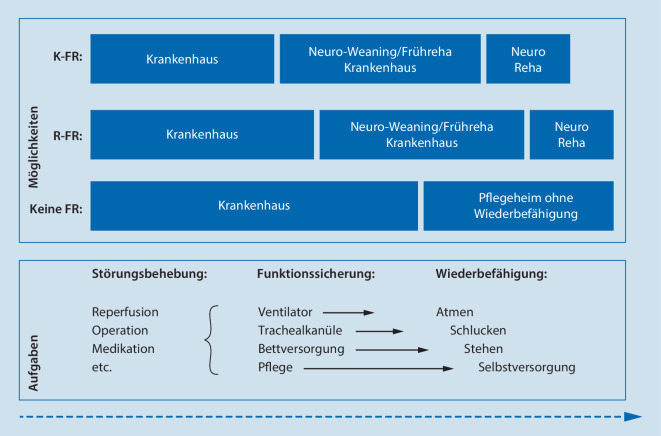

Krankenhäuser müssen auch kurzfristig reagieren. Die Zahl der anschlussrehabilitationsbedürftigen und in den Krankenhäusern fehlversorgten Patient*innen ist heute schon größer als zuvor und steigt weiter. Wenn Krankenhäuser nichts tun, riskieren sie in der Fallpauschalenlogik eine wirtschaftliche Abstrafung über ein Ansteigen der mittleren Verweildauer. Kliniken könnten Patient*innen leider weiter niederschwellig in Pflegeeinrichtungen entlassen. Dies ginge zu Lasten der Betroffenen und der Gesamtkosten des Systems. Ferner können Krankenhäuser sich eng mit integrierten Neurorehabilitationszentren abstimmen und Patient*innen in deren Krankenhausabteilungen zur neurologisch/neurochirurgischen Frührehabilitation oder, wenn Patient*innen noch beatmet sind, zum Neuroweaning verlegen. Schließlich können Krankenhäuser, wenn Nachversorgungsstrukturen fehlen, selbst Neurofrührehabilitation organisieren (Abb. 1).

Rehabilitation ist eine Querschnittsaufgabe im Gesundheitswesen (§ 1, SGB IX), und das Fallpauschalensystem verfügt über verschiedene OPS-Codes und damit Abrechnungsmöglichkeiten für Frührehabilitation im Krankenhaus. Der Aufbau einer Neurofrührehabilitation in einem Akutkrankenhaus ist schwierig und fiskalisch riskant, weil anspruchsvolle und von den Krankenkassen oft reklamierte Struktur- und Prozesskriterien gefordert sind. Auch ergeben sich nach der Fallpauschalenvereinbarung 2021, § 2, Abs. 1. in Fällen einer vorangehenden neurologischen Basisdiagnose Fallzusammenlegungen. Wenn aber Weiterversorgungskapazitäten fehlen, werden Akuthäuser aufgrund der Alters- und Morbiditätsentwicklung ihrer Patient*innen nicht umhinkönnen, diesen Schritt zu gehen. Frührehabilitation im Hause ermöglicht ihnen auch, Bewilligungsverzögerungen für Verlegungen von Patient*innen in die reguläre Rehabilitation finanziell abzupuffern. Denn solange Kassen keinen Rehabilitationsplatz liefern, müssen sie die Neurofrührehabilitation im Krankenhaus tagesbezogen weiterzahlen [4].

## Ausblick

Die Vermeidung verwahrender intensivmedizinischer Pflege durch Neurofrührehabilitation könnte die Gesundheitskosten wahrscheinlich um 1–2 Mrd. € pro Jahr senken [8]. Eine Abschaffung des Bewilligungsvorbehaltes für die Neurorehabilitation der Phase C würde bereits kurzfristig eine zusätzliche Summe von mehreren Milliarden Euro einsparen [23]. Konsequente Rehabilitation vor Pflege bei allen Krankenhausentlassungen einschließlich der zügigen Verfügbarkeit von Rehabilitationskapazitäten würde Pflegeversorgungskosten in noch höherem Umfang vermeiden [10]. In der Summe lägen die Einsparmöglichkeiten bereits bei einigen Prozentpunkten der Gesamtausgaben für stationäre Behandlungen.

Mit der weiteren anteiligen Zunahme älterer und kränkerer Patient*innen wird der postakute Versorgungsbedarf jedoch überproportional weiterwachsen. Wenn nicht gehandelt wird, könnten die Ineffizienzkosten bald in den zweistelligen Prozentbereich steigen – verbunden mit einer noch größeren unnötigen Bindung dringend benötigten Personals. Je früher wir aktiv werden, desto besser werden wir heutigen und zukünftigen Patient*innen gerecht.
